# Identifying extracellular vesicle populations from single cells

**DOI:** 10.1073/pnas.2106630118

**Published:** 2021-09-13

**Authors:** Jonas M. Nikoloff, Mario A. Saucedo-Espinosa, André Kling, Petra S. Dittrich

**Affiliations:** ^a^Department of Biosystems Science and Engineering, Eidgenössische Technische Hochschule Zürich, 4058 Basel, Switzerland

**Keywords:** extracellular vesicles, microfluidics, single-cell analysis

## Abstract

Extracellular vesicles (EVs) are constantly secreted from both eukaryotic and prokaryotic cells. EVs, including those referred to as exosomes, may have an impact on cell signaling and an incidence in diseased cells. In this manuscript, a platform to capture, quantify, and phenotypically classify the EVs secreted from single cells is introduced. Microfluidic chambers of about 300 pL are employed to trap and isolate individual cells. The EVs secreted within these chambers are then captured by surface-immobilized monoclonal antibodies (mAbs), irrespective of their intracellular origin. Immunostaining against both plasma membrane and cytosolic proteins was combined with highly sensitive, multicolor total internal reflection fluorescence microscopy to characterize the immobilized vesicles. The data analysis of high-resolution images allowed the assignment of each detected EV to one of 15 unique populations and demonstrated the presence of highly heterogeneous phenotypes even at the single-cell level. The analysis also revealed that each mAb isolates phenotypically different EVs and that more vesicles were effectively immobilized when CD63 was targeted instead of CD81. Finally, we demonstrate how a heterogeneous suppression in the secreted vesicles is obtained when the enzyme neutral sphingomyelinase is inhibited.

## Significance

Extracellular vesicles (EVs) are omnipresent in humans and contribute to the intercellular communication between cells. Their content, size, quantity, and surface markers depend on their cytosolic origin and the protein complex involved in membrane trafficking. Hence, vesicles may serve as valuable biomarkers, accessible via liquid biopsies. As of today, however, phenotype-specific EV subpopulations have not been clearly identified, mainly because EVs can be taken up and modified by other cells, hindering the heterogeneous EV production of individual cells. The proposed method enables robust classification of the EVs secreted by single cells, assessing their heterogeneity with respect to surface markers. In the future, this tool can be employed to assess the release and uptake dynamics of EVs in a controlled environment.

**Fig. 1.**
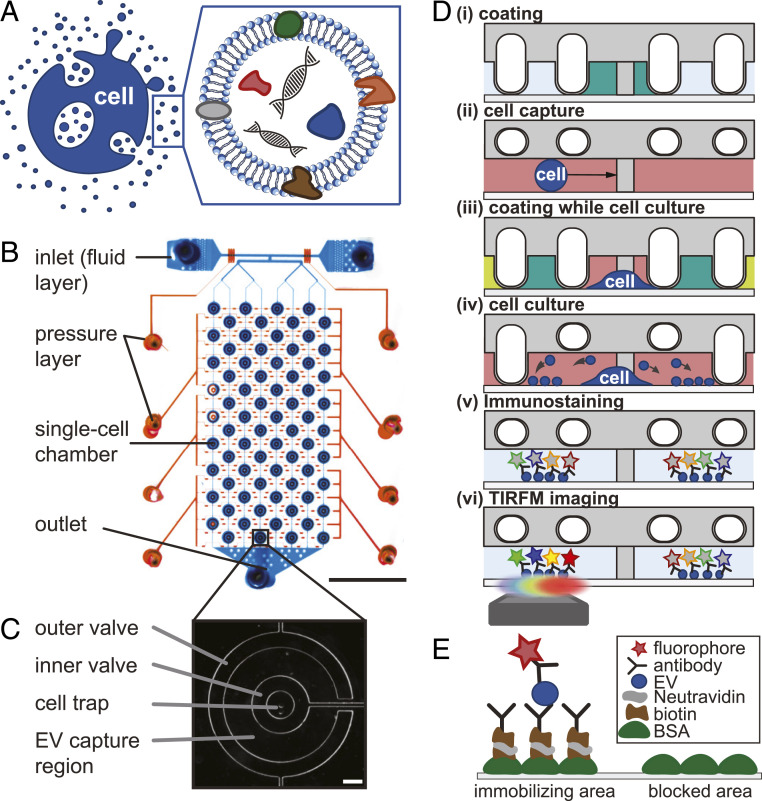
Schematic showing the motivation and experimental approach. (*A*) Carrying nucleic acids, proteins, and lipids, EVs are of major interest as potential biomarkers but cannot be properly distinguished from each other. Against the background of its biogenic origin, EVs are also seen as possible vehicles for pharmaceutical active compounds. (*B*) The PDMS-based two-layer microfluidic device describing the fluid layer (blue) with two inlets (*Top*), one outlet (*Bottom*), and the second, pneumatic control layer on top (red). (Scale bar 5 mm.) (*C*) Phase contrast image of a cell culture well. The outer valve encloses the area where EVs are immobilized. The inner valve allows isolating the hydrodynamically trapped cell during following coating. (Scale bar 100 µm.) (*D*) Functionalized surface with biotinylated BSA, NeutrAvidin, and biotinylated monoclonal antibodies to immobilize secreted EVs, which are then labeled with fluorescent-conjugated antibodies and imaged in four-color TIRFM. (*E*, *i*) Pneumatic valves on the microfluidic device enable spatially controlled surface functionalization. (*ii*) Single cells are entrapped at hydrodynamic traps. (*iii*) During cell culture, surface coating of EV immobilization in between the ring valves and BSA blocking against unspecific adsorption outside the outer ring valve are introduced. (*iv*) During cell culture, cells secrete EVs that are immobilized in direct proximity. (*v*) Secreted and immobilized EVs are immunologically stained. (*vi*) EVs are then imaged using TIRFM.

Mammalian cells secrete lipid bilayer–delimited compartments, commonly referred to as extracellular vesicles (EVs) (1). The heterogeneous entirety of these vesicles includes differences in membrane composition, encapsulated content, size, and cellular origin (2, 3). Vesicles are commonly divided into small vesicles of intracellular origin (40 to 200 nm), large microvesicles derived from the cell plasma membrane (200 to 1,000 nm), and larger apoptotic bodies (>1 µm). The smaller EVs, which are also referred to as exosomes, have received an increasing attention over the last 30 y. Since then, EVs have been shown to affect cells at diverse levels, from cell movement to immune modulation in stem cells (4, 5). The regulatory effect of exosomes, owed to the bioactive nature of their content (e.g., membrane proteins [Fig. 1*A*], nucleic acids, carbohydrates, and lipids), has boosted the identification of their biological purpose and function on physiological and pathological conditions, such as diabetes, cancer, and neurodegenerative diseases (6–9). Moreover, these vesicles have attracted interest as putative biomedical diagnostic tools or therapeutic drug vehicles (10–13).

While EVs are commonly enriched from cell culture supernatant using ultracentrifugation, they have also been concentrated from body fluids, like blood (14), urine (15), and saliva (16). The immunochemical features of EVs could also be exploited to isolate them, for instance, by targeting plasma membrane–integrated tetraspanins (2). Recent approaches use microfluidic techniques to enrich and/or isolate EVs using passive transport approaches [e.g., filtration (17), hydrodynamic manipulations (18), and deterministic lateral displacement (19)] or by applying external fields [e.g., acoustophoretic (14) and electrokinetic forces (20)]. The data collected using these techniques highlights the diversity of EVs/exosomes and the difficulty to identify or classify them accurately. Indeed, there is a unclarity about the usage and definition of terms like “exosomes” and “microvesicles” (2, 8, 21, 22). The generation and transport of EVs are heavily influenced by cell-to-cell communication, and their release and uptake mechanisms contribute to all levels of (patho) physiology (6, 7, 9).

However, it is not known how EVs secreted by a cell can be uptaken by other cells, modified, and released again into the bulk sample. To elucidate the biogenesis and secretion of EVs, it is of utmost importance to assess the statistics of single-cell EV secretion, without the potential interaction with other cells. This will give further insights into the most important cues of cell–cell interactions besides direct contact with other cells and the extracellular matrix and, most of all, signaling factors, which are commonly in the focus of single-cell studies.

Here, we introduce a microfluidic strategy to trap and culture single cells, to immobilize their secreted EVs, and to classify them by phenotype, independently of their cytosolic origin. A polydimethylsiloxane (PDMS)-based microfluidic device with arrays of two concentric, pneumatic valves is thus proposed. The sequential actuation of these valves enables the independent functionalization of the regions where cells and EVs will be incubated and immobilized, respectively. Although other platforms have been reported for the analysis of EVs derived from single cells (23), the proposed design allows for the analysis of EV population without any cross-contamination from other cells. In our method, antibody-based coating (for EV capture) is performed after individual cells are isolated in the central chambers. EVs secreted exclusively from the captured cell are efficiently captured in a small region between the two pneumatic valves. Therefore, the immobilization of EVs (suspended in the bulk cell culture) during the sample introduction is prevented, a critical challenge in the study of EVs secreted by single cells (23, 24). After the immunostaining of several proteins, immobilized EVs are imaged using four-color total internal reflection fluorescence microscopy (TIRFM) where TIRFM provides the necessary optical sensitivity and lateral resolution to approach the classification of single EVs into 15 unique phenotype populations. We demonstrate that EV subpopulations arise even at the single-cell level and can be identified by the proposed method.

## Results

### Description, Operation, and Validation of the Microfluidic Device.

Our focus is on collecting EVs secreted by single cells and performing their phenotype-specific classification (Fig. 1*A*). In addition to the isolation of single cells, this task requires a selective functionalization that promotes the immobilization of cells and EVs in different regions. Therefore, we designed a double-layered microfluidic device with two concentric valves as shown in Fig. 1*B*. The first layer comprises the fluid layer, where cells are introduced and captured by 72 PDMS posts which serve as hydrodynamic traps (Fig. 1*C*). The second (pressure) layer contains two round, donut-shaped valves per post, which can be lowered around the trapped cells to create isolated chambers (Fig. 1*C*). While the use of microfluidic valves to create cell culture chambers is common (25, 26), this two concentric valve design enables the regions where EVs will be immobilized to be functionalized once cells are isolated and protected by the central chamber, avoiding an initial cross-contamination.

The operation of the device can be described as follows. The area within the inner valve, which served as the cell cultivation area, was initially coated with fibronectin to promote cell adhesion (Fig. 1*D*, *i*). The cell suspension was then flushed in, and single cells were hydrodynamically trapped by the PDMS posts (Fig. 1*D*, *ii*). Once the cells were isolated and protected, the ring-shaped region between the valves was coated with antibodies against epitopes in the EV membranes, and the area outside the outer valve was coated with bovine serum albumin (BSA) (Fig. 1*D*, *iii*). The final cell culture chamber was achieved by closing and opening the outer and inner valves, respectively (Fig. 1*D*, *iv*). During culture, cells secreted EVs that were immobilized in the antibody-containing area between the valves (Fig. 1*D*, *iv*). After 24 h, the valves were reopened, fluorescently labeled antibodies were supplied to the EVs (Fig. 1*D*, *v*), and the system was imaged by four-color TIRFM (Fig. 1*D*, *vi*). The area outside the outer valve was coated with BSA to prevent unspecific adsorption (Fig. 1*E*).

**Fig. 2.**
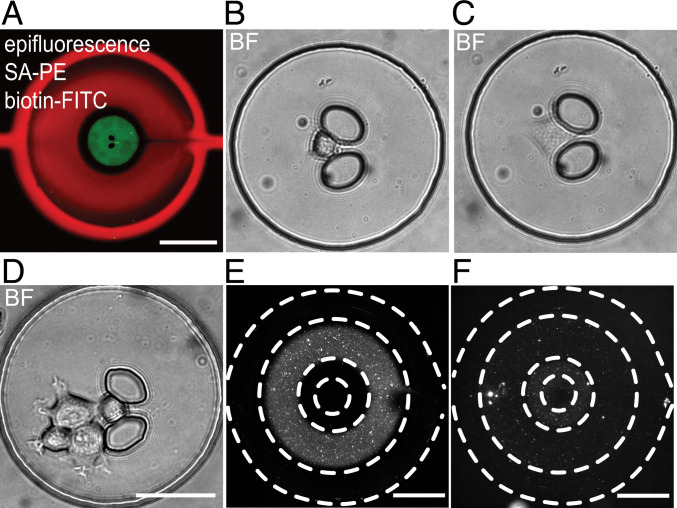
Spatial surface functionalization for cell incubation and vesicle immobilization. (*A*) Visualization of spatial surface functionalization in epifluorescence microscopy with false-colored PE (red) and FITC (green) after incubation in the respective areas, which were separated by actuated valves. The image shows a central green area hosting the hydrodynamic cell trap (two central black ellipsoids which are unstained, as they are plasma bonded to the microscopy coverslip). The cell cultivation area is surrounded by a black unstained ring, reflecting the PDMS–PDMS valve glass slide interface, and is enclosed by the immobilization area stained in red. (*B*–*D*) Brightfield images showing single (*B* and *C*) and multiple (*D*) incubated cells directly after trapping (*B*) and after <1 h (*C* and *D*). (Scale bar 50 µm.) (*E*) Having shown the principal surface coating, we incubated and immobilized biotinylated LUVs containing biotinylated lipids within the double valve–enclosed ring-shaped areas using BSA-biotin and NeutrAvidin and prevented their immobilization on the outside by passivating that area with 4 mass-% heat-denatured BSA. (*F*) Epifluorescent microscopy images of immobilized LUVs. (Scale bar 200 µm.)

**Fig. 3.**
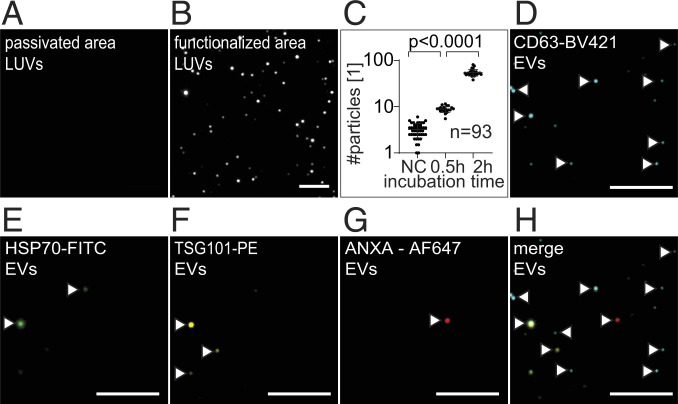
TIRFM showing functionalized surfaces for LUV and EV immobilization. (*A*) TIRFM images of BSA-passivated control areas, which do not immobilize EVs, next to the (*B*) BSA-biotin-NeutrAvidin–functionalized LUV-capturing area with a visible increase of detected LUVs. (Scale bar 5 µm.) (*C*) Quantifying immobilized LUVs per image shows increasing numbers of immobilized vesicles after 0.5 and 2 h compared to the control. The experiment was replicated twice with at least 93 measurements each. (*D*–*H*) TIRFM images of immobilized EVs after 24 h from MCF-7 cells excited in 405 (CD63-BV421, false colored in blue), 488 (HSP70-FITC, green), 561 (TSG101-PE, yellow), and 640 nm (ANXA5-Alexa Fluor 647, red) and merged. The signals are highlighted using white arrows. (Scale bar 5 µm.)

The capacity of the device to promote the adhesion and immobilization of cells and EVs, respectively, in adjacent, nonoverlapping regions, was initially assessed. To verify the differential functionalization, the surface between the two valves was coated with BSA-biotin and streptavidin-phycoerythrin (PE), while the area within the inner valves (the cell trapping area) was coated with BSA-biotin, NeutrAvidin, and biotin-fluorescein isothiocyanate (FITC). The resulting fluorescence of the nonoverlapping regions is shown in Fig. 2*A*. Next, the capacity of the fibronectin-coated regions to promote cell adhesion was inspected by flushing cells, trapping them, and verifying their adhesion (Fig. 2*B*–*D*). The ring-shaped regions, prepared with antibodies for vesicle capture, were tested using membrane-stained, biotinylated, large unilamellar vesicles (LUVs). After such regions were functionalized with BSA-biotin and NeutrAvidin, the LUVs were flushed into the chip, incubated for some time (Fig. 2*E*), and washed. The immobilized LUVs (Figs. 2*F* and 3*A* and *B*) were then inspected and enumerated (Fig. 3*C*). The regions blocked by BSA displayed ≤6 LUVs per image, while the immobilized vesicles at the nonblocked area increased along with the incubation time. After 0.5 h, ∼10 LUVs per image (two-sided Kolmogorov–Smirnov [KS] test, *D*_*KS*_ = 0.98, *P* = 2.58 × 10^−12^) were detected, an amount that increased 10-fold after 2 h of incubation (two-sided KS test, *D*_*KS*_ = 1.0, *P* = 5.27 × 10^−11^). These results demonstrate the blocking of unspecific vesicle binding in the BSA-passivated areas and the continuous immobilization of LUVs at the functionalized areas. Please note that, in TIRFM, the incoming excitation beam is reflected by the glass surface of the chip, creating an evanescent field within approximately 200 nm on top of the vesicle-immobilizing surface. Thus, nonimmobilized vesicles above the surface are not recorded.

### The Distribution of EVs Secreted in Each Well Correlates with the Incubated Number of Cells.

**Fig. 4.**
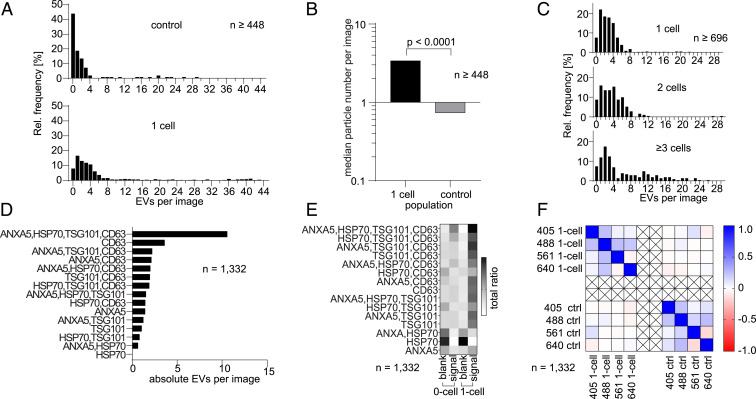
Detecting EVs at the single-cell level. (*A* and *B*) Relative frequency distribution of images taken from the EV-capturing areas after a 24-h culture of MCF-7 cells. The chips were functionalized with CD81 for immobilization. Areas feature higher signal densities in wells which were occupied by single cells than unoccupied control wells. (*B*) The relative frequency distribution shows increasing signal numbers (a larger median) compared to the control. (*C*) Relative frequency distribution representing the signal density of images in wells occupied with 1 cell, 2 cells, and 3 or more cells. (*D*) Corresponding to the relative increase of EV populations, we detected an absolute increase of EV number with up to 10-fold increase of the quadruple-positive EV populations. (*E*) Quantifying the categorical ratios of signals of EVs per image shows an increase of most EV populations in single-cell–occupied wells. (*F*) Testing for biased image analysis and creation of artifacts, we ran a two-tailed Pearson correlation coefficient analysis of single-cell–occupied wells and control wells showing the absence of analysis-based correlation biases.

The capacity of the platform to quantify EVs secreted from single cells was next assessed. The central chambers were coated with fibronectin, and secreted EVs were captured via CD81. After 24 h of cultivation, the cells and EVs were fixed with paraformaldehyde (PFA), and the whole device was treated with BSA to prevent unspecific adsorption. The EVs were then immunologically stained for target epitopes (i.e., CD63, HSP70 [70 kilodalton heat shock proteins], and TSG101 [tumor susceptibility gene 101]) as well as for phosphatidylserines (PS) in the outer membrane leaflet (a feature of distinct EVs) using Annexin V (ANXA5). The four-color TIRFM revealed the location of the immobilized EVs and their fluorescent signals, which were used to determine if they bind to any of the four supplied mAbs (Fig. 3*D*–*H*). The detected EVs per well were then enumerated: EVs were counted positive if they bound at least one of the antibody conjugates as shown in Fig. 3*H*. To determine whether single-cell–containing wells would reveal more EVs than empty wells, the distribution of the signals identified as vesicles was compared for both cases. Fig. 4*A* shows such vesicle distributions after 24 h of incubation in which >1,000 TIRFM images were analyzed. The EV frequency distribution in the single-cell wells is different to that of the empty wells (two-sided KS test, 1 versus 0 cells, *D*_*KS*_ = 0.51, *P* < 2.2 × 10^−16^). The distribution core for the empty wells is located in the low EVs per image region, with 45% of them having zero signals identified as vesicles compared to 8% of the singly occupied wells. Signals detected in the nonoccupied wells could be attributed to unspecific background adsorption. However, such signals are scarce, as only 26% of the nonoccupied wells show more than one EV per image compared to 76% of the wells with single cells. Taking the median as a representation of central tendency, the single-cell–occupied wells encompass a threefold higher signal than nonoccupied control wells (Fig. 4*B*).

When the single-cell wells are compared with those having multiple cells, a progressive “flattening” in the distribution of EVs per image was observed as the number of cells increases (Fig. 4*C*). The EVs distribution for the wells with one cell shows a prominent peak at approximately three EVs per image (82% of the wells contain one to five EVs per image) and light tails (only 7% of the wells contain greater than six EVs). The wells holding a pair of cells, in contrast, show a less-localized peak (83% of the wells contain one to nine EVs per image). The data analysis reveals a flatter distribution for wells containing three or more cells. The prominent peak observed at approximately three EVs per image for the single-cell wells is now spread out over a larger range, with 83% of the wells containing 1 to 16 EVs. In addition, the distribution tail becomes heavier, as a larger proportion of wells contain >10 EVs (4, 6, and 27% for the wells with one, two, and three or more cells, respectively). The proposed technique is therefore sensitive enough to differentiate between the number of EVs secreted by one, two, and three or more cells (two-sided KS tests: 1 versus 2 cells, *D*_*KS*_ = 0.25, *P* = 2.7 × 10^−7^; 1 versus 3+ cells, *D*_*KS*_ = 0.31, *P* = 6.4 × 10^−13^; and 2 versus 3+ cells, *D*_*KS*_ = 0.21, *P* = 7.7 × 10^−4^). The nonlinear nature in the EVs secreted as a function of the number of cells per well evinces the complex dynamic behavior behind the uptake and release of EVs (27, 28). However, further experiments with paired cells are required to elucidate the effects of mixed EVs (secreted by different cells) and the uptake-release dynamics. The results presented in this manuscript construct toward this goal by enabling the assessment of the statistical behavior of individual cells.

### Cells Secrete Phenotypically Heterogeneous EV Populations.

Next, our platform was used to analyze the phenotype of EVs secreted by single cells. Antibodies against HSP70, TSG101, and CD63 were supplied to the EVs in addition to ANXA5 (against PS). After washing, the TIRFM analysis revealed that, although many EVs tested positive for the four markers, not all EVs showed signals above the detection threshold in the four channels. The population occurrence is introduced in Fig. 4*D* and *E*. The quadruple-positive vesicle subpopulation (ANXA5^+^HSP70^+^TSG101^+^CD63^+^) was the largest one, followed by the double-positive CD81^+^CD63^+^ subpopulation (Fig. 4*D*). The observed distribution of the EV population underlines the heterogeneity of their secretion and reinforces the hypothesis that these subpopulations are secreted at different frequencies (29). Moreover, these results emphasize the need for several markers to adequately identify EVs or exosomes. To test if there exists any correlation between the detected fluorescent signals, in which case artificial similarities between EV populations would be introduced, the Pearson’s correlation coefficient was calculated for all fluorescent channel pairs (Fig. 4*F*). The correlation matrix shows no strong positive or negative correlations between the detected signals, neither in the control nor in the single-cell–occupied wells.

### The Immobilization Strategy Affects the Population Composition.

**Fig. 5.**
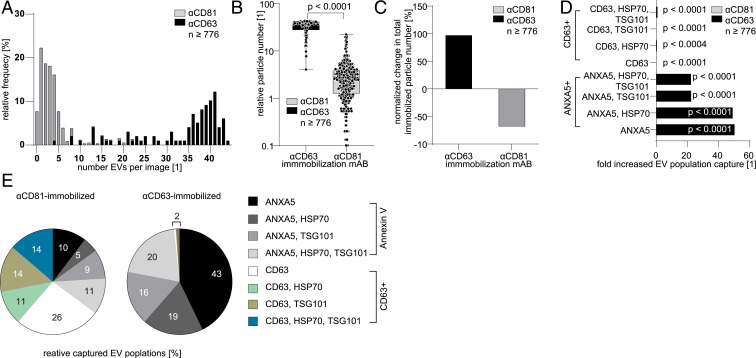
Immobilization technique affects immobilization efficacy and detected population compositions. (*A*) Histogram and (*B*) scatter dot plot showing numbers of immobilized vesicles using CD81 or CD63 for EV immobilization within 24 h from single MCF-7 cells. The EVs per image increase when immobilizing EVs with CD63 instead of CD81. (*C*) Bar plot showing the normalized change in vesicle numbers upon immobilization via CD63 or CD81. (*D*) Bar plot showing the fold changes of EVs clustered into ANXA5^+^ and CD63^+^ EV populations. Upon immobilization with CD63, ANXA5^+^ populations increase in number, and CD63^+^ vesicle numbers decrease (tested against CD81-immobilized EVs). (*E*) Color-coded pie charts summarizing the relative composition of EV populations when immobilized with CD81 (*Left*) or CD63 (*Right*) corresponding to ANXA5^+^- and CD63^+^- clustered EV populations.

It is under current discussion if there are membrane-integrated proteins that are specific for EVs. (2, 27) “Exosome-specific” reported markers include tetraspanins (e.g., CD9, CD63, and CD81), HSP70, and flotillin-1 (2, 27, 30). A main difficulty for choosing a single confirmed marker is the lack of knowledge on the complete biogenesis and pathways of EVs, including exosomes, in cells. Therefore, the proposed platform was employed to assess and compare the immobilization efficiency of two mAbs against the commonly cited class of tetraspanins: CD63 and CD81 (Fig. 5). The analysis of ∼500 TIRFM images per channel showed that, after 24 h incubation of single MCF-7 cells, the distribution of signals detected in the CD63-mAb–functionalized wells is significantly larger (two-sided KS test, *D*_*KS*_ = 0.94, *P* < 1 × 10^−15^) than those detected in the CD81-mAb wells (Fig. 5*A*). The distribution core for the CD81-immobilized EVs is centered at approximately three EVs per image, with 29% of the images revealing less than or equal to one vesicle. Another 29% contain four to eight vesicles and only 4% up to 22 EVs per image. In contrast, the peak of the CD63-positive distribution is located ∼40 EVs per image. More than half (57%) of all images show 20 to 40 EVs, with only 10% containing 10 to 20 EVs, and 4% show <10 EVs per image. The proposed combination of the microfluidic device and four-color TIRFM was able to characterize a 10-fold increase in the 95th percentile of each functionalization strategy (Fig. 5*B*). These results evince that the choice of marker introduces a bias in the measurements (as is the case in all affinity positive selection methods) that should be considered during analysis.

To determine the influence of the immobilization approach on the phenotypical composition of EVs, the occurring EV populations were clustered considering the different phenotypes. The occurrence rate of CD63^+^ and ANXA5^+^ EVs revealed that the CD81-immobilized EV populations were lower in ANXA5^+^ vesicles compared to the CD63-immobilized EVs (χ^2^-tests for detecting differences after treatment; the *P* value was <3.574 × 10^−4^ in all cases; the exact values for each comparison can be found in *SI Appendix*). We found that the relative abundance of EVs immobilized via anti-CD63, and carrying ANXA5, increased up to 20 and 50 times, depending on the respective phenotype populations (Fig. 5*D*). The same EVs, however, comprised no secondary detectable CD63 antigen. The loss of codetected secondary CD63 (when using CD63 instead of CD81 for the immobilization) occurred homogeneously throughout the CD63-positive populations. The overall change in the composition of EV populations is visualized in Fig. 5*E*, where the phenotypic EV populations are clustered by being positive for CD63 or ANXA5, independently of the immobilization mAb. When CD81 was employed, the CD63^+^ and ANXA5^+^ populations accounted for 65 and 35% of all vesicles (Fig. 5*E*). In contrast, when EVs were immobilized using the anti-CD63 mAb, the EV populations that were positive for CD63 diminished throughout all CD63^+^ populations to 2% (Fig. 5*D* and *E*). The capture of EVs via CD63, however, resulted in elevated levels of PS-rich EVs to about 98% (Fig. 5*D* and *E*).

### Quantifying the Down-Regulation of EV Secretion by the Neutral Sphingomyelinase Inhibitor GW4869.

**Fig. 6.**
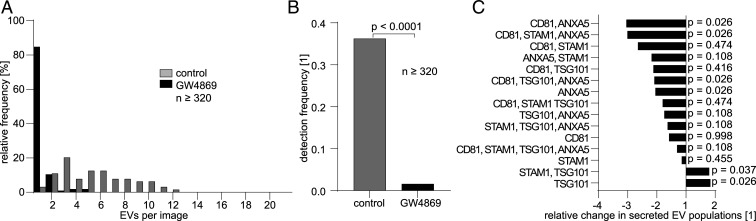
Sphingomyelinase inhibitor GW4869 diminishes secretion of some EV populations. (*A*) Histogram of the relative frequency distribution of secreted EVs from single MCF-7 cells incubated for 24 h in 5 µM GW4869 and without (control) show reduced vesicle secretion when GW4896 is present. (*B*) The relative detection frequency of EVs diminishes upon incubation with GW4869. (*C*) The population-wise analysis of EV secretion shows up to threefold relative reduction under GW4869 incubation for EVs which are CD81^+^ ANXA5^+^ (and CD63^+^) compared to the control and a relative up-regulation on TSG101^+^ and TSG101^+^ STAM1^+^ EVs. The *P* values for all pairs (compared to the control) are indicated in the figure.

The effect of the enzymatic inhibitor GW4869, which down-regulates the enzyme neutral sphingomyelinase, was studied using our platform. It has been previously reported that exosome numbers drop upon blockage using GW4869 (31), as this inhibitor participates in the invagination process into multivesicular bodies (32). We thus supplied a 5-µM solution of GW4869 to the isolated cells and analyzed the changes in the composition of the secreted EVs. If whole populations are compared, then a decrease in the number of secreted vesicles for the cells treated with the inhibitor is observed (two-sided KS test, *D*_*KS*_ = 0.87, *P* < 1 × 10^−15^) (Fig. 6*A* and *B*). A deeper examination reveals, however, that the observed population decrease is caused by only a few subpopulations being down-regulated (Fig. 6*C*). The effect of GW4869 was assessed by comparing the number of vesicles produced with and without the treatment for every subpopulation, and we focus on those showing significant differences (the *P* values and D-statistics for all comparisons are given in *SI Appendix*). After EVs were immobilized via CD63, the CD81^+^ANXA5^+^ and CD81^+^STAM1^+^ANXA5^+^ subpopulations were found to exhibit the strongest down-regulation (threefold decrease; two-sided KS test, *D*_*KS*_ = 0.83, *P* = 0.026). These classes were followed by CD81^+^TSG101^+^ANXA5^+^ and ANXA5^+^, showing a twofold decrease (two-sided KS test, *D*_*KS*_ = 0.83, *P* = 0.026). In contrast, the STAM1^+^TSG101^+^ and TSG101^+^ classes, both ESCRT dependent, showed a relative increase in vesicle production. These results seem to indicate a strong effect on vesicle secretion for those rich in PS, CD81, STAM1, or TSG101 and less impact on those secreted via the ESCRT-dependent pathway (31, 33).

## Discussion

Mammalian cells secrete EVs, which differ in their morphology and biochemical composition. Given the large heterogeneity and small size of these vesicles, it is extremely challenging to identify them, differentiate their phenotypes, and track back their biogenesis. Therefore, the enrichment and classification of EVs usually rely on their physical properties, like density and diameter. In this manuscript, we present a microfluidic strategy to immobilize EVs secreted by single cells and classify them by phenotype. While the creation of individual incubation compartments is a common approach in microfluidic devices (26, 27), the proposed double-valved design enables the selective functionalization of the ring-shaped area next to the cultured cells. In this manner, EVs are immobilized and analyzed in situ, preventing their rupture or other changes as usually occurs when an external enrichment step is required (Fig. 1*D*). More importantly, the design allows this ring-shaped region to be functionalized with antibodies after the cells are introduced and protected. This prevents any contamination by EVs immobilized when the bulk cell culture supernatant is introduced, a common issue in single-cell studies dealing with EVs (24).

After EV immobilization, the immunostaining of several proteins and the imaging using four-color TIRFM facilitated their enumeration per surface area in which TIRFM provided the necessary optical sensitivity and lateral resolution to approach the phenotype-specific classification of single EVs. Several markers for membrane-integrated and cytosolic proteins were chosen for the immobilization and immunostaining of EVs, based on their use in previous studies for the characterization and identification of EVs (2, 23). However, the cytosolic origin of the EVs was not contemplated in the analysis.

Lipophilic dyes such as DiO can be, in principle, used as a control to stain and enumerate EVs (34–37). However, morphological artifacts were observed for DiO-stained EVs during the TIRFM imaging (*SI Appendix*, Figs. SI5 and SI6). Additional control experiments were performed with the three lipophilic dyes (Octadecyl Rhodamine B Chloride [R18], 9-(Diethylamino)-5H-benzo[a]phenoxazin-5-one [Nile Red], and PKH26 [*SI Appendix*, Fig. SI7]). They all showed significant larger unspecific background adsorption, which prevents reliable EVs quantification in wells that are occupied by one or more cells. Therefore, we abandoned the use of unspecific labels.

The largest population of EVs was positive for all the selected markers, confirming the immobilization of EVs, including exosomes, on the microfluidic device. These results demonstrate, in addition, that the antibody stains are not sterically hindered. In contrast, the populations carrying only one, two, or three epitopes demonstrated the heterogeneity of EVs with respect to their origin and/or size. These results show a bias in the measurements (with respect to the chosen antibody) that needs to be considered before results can be generalized.

As expected, and in accordance with most reports on EVs (30), tetraspanins were detected. Besides tetraspanins, which represent membrane-integrated epitopes, STAM1 and TSG101 were also detected. The former are components of the ESCRT-0 and -I subunits, involved in EV trafficking (38). Moreover, EVs carrying HSP70 [a member of the heat-inducible and constitutively expressed HSP family that has been reported in EVs (23)] were detected. PS-rich vesicles were also identified (via ANXA5) to confirm the intracellular origin of the EVs (2, 39). To confirm that we are capable of staining cytosolic proteins (i.e., luminal-located proteins) due to the membrane-perforating effect of PFA and that mAbs can thus cross the lipid bilayer, the cytosolic localization of PS via ANXA5 (*SI Appendix*, Fig. SI2*A*) and CD63 (*SI Appendix*, Fig. SI2*B*) was validated.

Due to the large incidence of EVs carrying the CD63 epitope, the capture efficiency of EVs using anti-CD81 and anti-CD63 was compared. The number of immobilized EVs was significantly larger when anti-CD63 was employed, a result in agreement with previous works that compares the capturing of EVs via tetraspanins EVs (40, 41), indicating a lower number of vesicles bearing CD81 epitopes. The larger capture efficiency can also be related to a larger binding constant for the anti-CD63 antibody (42).

Further experiments on the immobilized EVs revealed that the proportion of subpopulations differed. For instance, the EVs immobilized by anti-CD63 no longer exhibited CD63 epitopes on the free (surface-averted) side. We speculate that either free diffusion (within the lipid membrane of CD63) toward the surface-bound antibodies depleted the presence of CD63 on other areas of the EV membrane or that the stochastic distribution of tetraspanins-enriched microdomains (TEMs) resulted in an immobilization of EVs (via CD63) and the presentation of potentially CD63-negative (but CD81-positive) TEMs, as described by Nydegger et al. (43), Deneka et al. (44), and Charrin et al. (45, 46) When the immobilization antibody was changed from CD81 to CD63, a distinct change in the population composition of EVs was observed, leading to an emphasized fraction of ANXA5^+^ vesicles. Similar results about ANXA-positive EVs were reported earlier by Jeppesen et al. (47). ANXA5 is a commonly used marker for the detection of early apoptosis and for changes in the asymmetry of membranes due to apoptosis (48). However, ANXA5 was employed here to detect phosphatidyl-enriched EVs. Thus, EVs of cytosolic origin, like apoptotic cells, were excluded from the analysis (using brightfield microscopy). We therefore consider the possibility that EVs secreted by MCF-7 cells and immobilized via CD63 are enriched in phosphatidylserine, a result that may indicate that the CD63^+^PS^+^ subpopulation has a cytosolic origin (39, 49, 50).

The inhibition of neutral sphingomyelinase by GW4869 as a negative control is often employed to identify specific epitopes for EVs and the physiological activity of EVs and alleged subpopulations (e.g., on other cell types) (31, 51). GW4869 acts on the ESCRT-independent pathway of exosome secretion (52, 53), down-regulating exosome production. Hence, the influence of this enzymatic inhibitor at the single-cell level was quantified. Although the data analysis indicated a generalized down-regulated EV secretion, only some populations were strongly affected. Vesicle production in the CD81^+^ANXA5^+^ and CD81^+^ANXA5^+^STAM1^+^ populations was largely suppressed, while production was up-regulated in the TSG101^+^ and STAM1^+^TSG101^+^ populations, an observation that has been reported under similar conditions for other cell types (54).

In summary, the proposed microfluidic device robustly isolates individual cells and prevents any cross-contamination because of the introduction of the antibodies after cell immobilization. Immunostaining in combination with four-color TIRFM allows for a robust counting and classification of the EVs truly secreted by single cells. Our findings indicate that the immobilization strategy heavily affects the phenotypical compositions of the detected EVs and that the negative regulatory effect of GW4896 has a nonhomogeneous effect on the EVs. The ability to track variations in the phenotypical EV unit, with respect to the applied immobilization approach, will allow, in the future, to link metabolic and genetic conditions to the formation and secretion pathways of EVs and, hence, to cell-to-cell communication at the single-cell level.

## Materials and Methods

### Mammalian Cell Culture.

Michigan Cancer Foundation (MCF)-7 cells were maintained in standard continuous cell culture conditions in Dulbecco’s Modified Eagle’s Medium (DMEM) supplemented with 1 g ⋅ L^−1^ glucose, pyruvate, and 10 vol-% fetal bovine serum (both Thermo Fisher Scientific) at 37 °C, 5 vol-% CO_2_, and a relative humidity of 95%. Cells were trypsinized (0.05% 2,2’,2’’,2’’’-(Ethane-1,2-diyldinitrilo)tetraacetic acid [EDTA], Thermo Fisher Scientific) and passaged in 1:5 ratios twice a week. On-chip culture conditions included 1× Penicillin-Streptomycin (Thermo Fisher Scientific) and 2 vol-% exosome-depleted fetal bovine serum (System Biosciences). GW4869 was purchased from Sigma-Aldrich and diluted according to the distributor recommendation.

### Microfluidic Device Fabrication.

The PDMS devices were fabricated using a 10:1 mass ratio of Sylgard 184 Silicon Elastomer Base and Curing Agent (Dowsil, formerly Dow Corning Midland). The mixture was degassed until visually bubble free. Next, 40 g mixed PDMS was poured onto the master mold containing the pressure layer, and the mold was placed at 80 °C for >2 or 24 h. Later, 5 g PDMS were spin coated (20 s at 500 rpm and 40 s at 2,800 rpm) on the fluid layer mold and cured at 80 °C for 1 h. This process resulted in a flexible membrane of approximately 25 µm thickness. After curing, the pressure layer was peeled off the master mold. Pressure inlet holes were then punched using a 1-mm biopsy puncher (Integra). For bonding the pressure layer onto the fluid layer, 2 to 3 mL curing agent was poured onto a blank 4-in silicon wafer and spin coated at 6,000 rpm for 1 min. The punched-pressure layer devices were dropped onto the curing agent–coated blank silicon wafer, peeled off, and manually aligned onto the fluid layer. The edges of the combined layers were sealed with degassed PDMS mixture. The assembled chips were cured at 80 °C for 2 h.

After curing, the assembled PDMS layers were peeled off the fluid mold, and inlets/outlets were punched using a 1.5-mm biopsy puncher (Integra Miltex York). The assembled devices were cleaned using adhesive tape. In parallel, 1 g PDMS mixture was spin coated on No. 1 microscopy glass slides (6,000 rpm for 60 s) to cover them with a 10-µm-thick PDMS layer. The PDMS layer was then allowed to reflow for over 30 min at room temperature (RT) before being cured overnight at 80 °C. The double-layer PDMS devices and PDMS-coated microscopy glass slides were plasma activated (PDC-32G, Harrick Plasma) at ∼0.77 mbar for 45 s (18 W), and bonded together. The glass-bonded devices were put on a heating plate at 100 °C for 10 min and stored at RT before usage.

### Preparation and Surface Functionalization of the Device.

All devices were filled with milliQ water before use, by spinning them at 800 g for 10 min, and incubated at 37 °C, 95% relative humidity, and 5 vol-% CO_2_ for >30 min. The devices were then connected via a polytetrafluoroethylene tubing (PKM SA), a silicon tubing (Gobatec) and polyether ether ketone microfittings to 10-mL syringes (Becton Dickinson). The syringes were loaded on syringe pumps (either NE-1002X-ES, World Precision Instruments, or NanoJet, Chemyx, Inc.). The pressure channels were connected via bent metal pins to a silicon tubing, which was connected to pressurized air via a manual, customized control unit (Cole-Parmer). Devices were flushed with sterile phosphate-buffered saline (PBS) without (w/o) Ca^2+^ and Mg^2+^ (Sigma-Aldrich). To promote cell adhesion, each cell-capturing area on the entire devices was flushed with 100 ng ⋅ mL^−1^ fibronectin (Sigma-Aldrich) and incubated for 30 min (inner valves closed) and then flushed with PBS w/o Ca^2+^. After incubation, the chip was washed with exosome-depleted fetal calf serum–supplemented medium (2 vol-%, 1× Penicillin-Streptomycin, 1 g ⋅ L^−1^ glucose DMEM [exoPSFDMEM]). Next, trypsinized and strained (35 µm) cells were flushed in, trapped, and incubated at 37 °C, 5 vol-% CO_2_. After cell trapping, the inner ring valves were actuated. In order to minimize adverse effects for the isolated cells, such as the accumulation of metabolic compounds or the arise of nutritional stress, which may affect the biogenesis and secretion of EVs, all incubation steps were limited to 24 h. The medium per cell was ∼5.5 × 10^−2^ µL per cell, corresponding to the volume of fresh medium per cell at a confluency of 60% during standard cell culture conditions. In comparison, the medium per cell would be about 3.3 × 10^−2^ µL per cell after 72 h of incubation, equivalent to a 100% confluency. The EV-capturing area was incubated with 2 mg ⋅ mL^−1^ biotinylated BSA (Thermo Fisher Scientific) for 30 min, followed by 100 g ⋅ mL^−1^ Neutravidin and 5 ng ⋅ mL^−1^ biotinylated conjugates of the respective monoclonal immobilization antibodies, anti-CD63 or anti-CD81 (BioLegend), for 30 min. After incubation of the area for EV capture, the device was flushed with exosome-free exoPSFDMEM.

### Large Unilamellar Vesicle Production.

For preparation of biotinylated LUVs, a 20-mM lipid solution, consisting of 95 mol-% 1-palmitoyl-2-oleoyl-glycero-3-phosphocholine (16:0 to 18:0) and 5 mol-% 1,2dioleoyl-sn-glycero-3-phosphoethanolamine-*N*-(biotinyl) (DOPE) in chloroform (Sigma-Aldrich) was prepared. DOPE, a biotinylated lipid, was introduced into the LUV membrane to make the LUV model as similar as possible to EVs. All lipids were purchased from Avanti Polar Lipids, Inc.. A dried lipid film in a glass flask was created from 10 mL lipid solution using a rotational evaporator to remove the chloroform (10 min at 200 mbar and RT followed by 15 min at 5 mbar and RT and 1 h desiccated at 20 mbar and RT). The lipid film was then rehydrated in PBS. The suspension underwent 10 freeze and thawing cycles in liquid nitrogen and a water bath (T = 40 °C). The created vesicles were then extruded in a miniextruder (Avanti Polar Lipids, Inc.) using a polycarbonate membrane with pore sizes of 100 nm (Sigma-Aldrich). The size distribution of the vesicle suspension was determined in a Zetasizer Nano ZSP (Malvern Panalytical GmbH). The coating of the microfluidic chips with BSA-biotin and NeutrAvidin resulted in a similar approach to the targeting of EVs via BSA-biotin, Neutravidin, and biotinylated mAbs.

### Immunocytochemistry.

Cultivated cells were washed in PBS with Ca^2+^ and Mg^2+^, fixed in 4 vol-% paraformaldehyde in PBS with Ca^2+^ and Mg^2+^ (pH 7.2), followed by blocking in 4 mass-% heat shock–denatured BSA and stained for respective epitopes in 0.1 mass-% heat shock–denatured BSA in PBS with Ca^2+^ and Mg^2+^. The antibodies were purchased from Biolegend UK Ltd (biotinylated anti-CD63 and anti-CD81 [both 5 μg ⋅ mL^−1^], anti-CD63-PerCPCy5.5, anti-CD81-PE, ANXA5-Alexa Fluor 647) and from Santa Cruz Biotechnology (HSP70 [HSC70/HSPA8]–FITC, STAM1-FITC, TSG101PE).

### Data Analysis and Statistics.

Several experiments were performed to collect TIRFM images from empty wells (0-cell, *n* ≥ 448) and wells containing single cells (1-cell, *n* ≥ 448), two cells (2-cell, *n* ≥ 696), and three or more cells (+3-cell, *n* ≥ 696). After the image analysis (described in detail in *SI Appendix*), the frequency distribution of the detected signals per image was computed for each case; each image represents an area of 4,356 µm^2^. The platform was considered to be sensitive if the frequency distribution of the detected signals was significantly different between all pair combinations in the set {0-cell, 1-cell, 2-cell, +3-cell}. The difference in the distributions was tested using the two-sided KS test. However, the KS test assumes no ties and is known to produce conservative *P* values when applied to discrete data. To account for this, we employed a bootstrap approach with *n*_*b*_ =10,000 samples to compute the null distribution of the D-statistic and provide a corrected *P* value (55). In all cases, the *P* value was below *P* ≤ 7.7 × 10^−4^, showing that there is enough information to reject an equality in the signal distributions. Moreover, permutation tests of equality were conducted by approximating the distributions with densities (56), with all combinations showing a *P* value of approximately zero. While noise signals were detected at the empty wells (i.e., unspecific adsorption), almost half (45%) of the processed images showed no signals at all. In contrast, only 7% of the images obtained from wells containing single cells showed no signals. The same proportion (7%) was found in the wells containing two cells and in those containing three or more cells, showing the reproducibility of the proposed workflow. The proposed approach immobilizes a subset of the vesicles secreted by cells at the surface of the isolation chambers, and TIRFM is then employed to detect the ones attached to the bottom substrate (see *Description, Operation, and Validation of the Microfluidic Device*). We expect this bottom substrate attachment to have a stochastic nature. Therefore, the distance covariance test of independence was employed to confirm the independence between images sampled from the same well (57). A bootstrap approach with *n*_*b*_ = 10,000 replicates per case was followed. The *P* value was, in all cases, larger than 0.001 (distance covariance *D*_*Cov*_ > 1,932), indicating that there is not enough information to refuse the no association between variables, except in some isolated situations (less than 0.1 and 1.0% of the analyzed cases for significance levels of 0.001 and 0.01, respectively).

## Supplementary Material

Supplementary File

Supplementary File

## Data Availability

The derived datasets generated during and/or analyzed during the current study are available at Dryad, https://doi.org/10.5061/dryad.dz08kprz5 (58). The image processing codes are available as supplementary files and online at Zenodo, https://doi.org/10.5281/zenodo.5211393 (59).
