# Supplementary material for: Identifying extracellular vesicle populations from single cells
Source: Proc Natl Acad Sci U S A. 2021 Sep 13;118(38):e2106630118. doi: 10.1073/pnas.2106630118 (PMC8463870; doi:10.1073/pnas.2106630118)
Supplement: Supplementary File [file pnas.2106630118.sapp.pdf]

Supplementary Information

Identifying extracellular vesicle populations from single cells

Jonas M. Nikoloff, Mario A. Saucedo-Espinosa, André Kling and Petra S. Dittrich*

Department of Biosystems Science and Engineering, ETH Zurich, Basel, Switzerland

Corresponding author

Petra S. Dittrich

Mattenstrasse 26

CH-4058 Basel, Switzerland

e-mail: petra.dittrich@bsse.ethz.ch

phone: +41 61 387 33 10

Methods.

Electron microscopy. Scanning electron microscopy was conducted by the Imaging Facility of the University of Basel and the Center for Cellular Imaging and Nanoanalytics using an SEM Hitachi S4800 (K.K. Hitachi Seisakusho) on 4 nm-thick platinum coated round glass slides (No. 0, VWR international). Transmission electron microscopy was done by applying 5 µL of the 100k-EV pellet from MCF-7 cell culture supernatant onto a Parlodion-coated copper grid 400, which had been glow-discharged for 30 s. The sample was incubated for 1 min, blotted, and washed three times with water before being stained with 2% uranyl acetate for 10 min. The samples were imaged with a Tecnai G2 Spirit 120 kB TEM microscope (Thermo Fisher Scientific) equipped with an EMSYS Morada camera (Emsys Embedded Systems GmbH).

Master mold fabrication. Silicon master molds for the PDMS chip replication were produced in a standard cleanroom environment. The mold designs were enlarged to 101.16% of their actual size to account for shrinkage of the cured PDMS. SU-8 photoresist (Microchem) was spin-coated on a 4-inch silicon wafer to a final thickness of 20 µm. The resin was baked and UV-exposed (160 mJ cm^{-2}, i-line) using an MA-7-mask aligner (Süss Microtec, Germany). A foil mask (Selba, Switzerland) was used to define the control and fluid layers in the molds. The master molds were baked at 65 °C for 5 min and at 95 °C for 12 min, developed, and finally hard-baked at 120 °C for 45 min before being treated with 1*H*,1*H*,2*H*,2*H*-perfluorodecyltrichlorosilane (ABCR, Karlsruhe, Germany). The molds were finally coated with polytetrafluoroethylene (PTFE) using 0.1 vol-% poly[4,5difluoro-2,2-bis(trifluoromethyl)-1,3-dioxole-cotetrafluoroethylene] in FC-40 fluorinated oil (all Sigma-Aldrich).

Fluorescence microscopy. The devices were imaged in brightfield mode with an LED illumination system (CoolLED Ltd., Andover, UK) at 20-fold magnification (NA = 0.75) and a Hamamatsu Orca flash 4.0 V2 CMOS camera with a sensor size of 2044×2048 pixels during

on-chip cell culture. Epifluorescent images were acquired using a Spectra X LED unit (Lumencor Inc., Beaverton OR, USA) for excitation and optical filters and dichroic mirrors for FITC (Semrock FF01-475/28 nm, SemrockFF03-525/50 nm, Chroma T495lpxr), and for phycoerythrin (PE) (Semrock FF01-549/15, Semrock FF01593/40, Semrock FF562-Di03) (Chroma Technology GmbH, Olching Germany, IDEX Health & Science, LLC Rochester, NY, USA). For EV detection, the built-in VisiScope (VS) TIRFM System consisting of a Nikon Ti2 E inverted microscope with a motorized stage, a VS Laser Modul 1865 with Laser- Merge and VS Phase switcher for excitation at 405 nm, 488 nm, 561 nm and 640 nm was used. The system was equipped with a Quad Band filter cube (TRF89901, Chroma Technology GmbH, Olching, Germany) and additional emission filters of 460/50 nm, 525/50 nm, 609/54 nm, and 700/75 nm (Chroma Technology GmbH, Olching, Germany, IDX Health and Science, Rochester, NY, USA).

Images were taken with a PRIME-95B Back-illuminated scientific CMOS camera (Teledyne Photometrics, Tucson AZ, USA, Visitron Systems GmbH, Puchheim, Germany). TIRFM imaging was conducted using an immersion oil Nikon CFI Apochromat TIRF 100× objective (NA = 1.49). Imaging was controlled by Visitron VisiView.

Image analysis. A custom MATLAB script for the analysis of the TIRFM images was developed. The script uses a JAVA library for bio-formats to access the nd-files. Vesicles were detected as particles. Outlier detection was performed by thresholding the signals using the median value and the median absolute deviation as a dispersion metric[56]. After outliers had been detected, the images were segmented into binary masks, and merged together. The binary masks were then prepared using several image processing techniques, namely hole filling, erosion, and dilation. Next, detected particles with an area above 1,500 pixels^2 were removed. Since the camera had a sensor of 1,200 pixels and a 2×100 magnification was employed, each pixel of an image corresponded to 55 nm of the microfluidic chip. In the case where the detected particles were semicircular, particles with a diameter larger than 1.2 µm

were excluded. Overlapping signals underwent a watershed segmentation and were merged together. The signals in every fluorescent channel were then filtered using the median value plus at least two median absolute deviation units as a lower bound for signal discrimination. This process resulted in a binary attribute for every fluorescent channel: either the signal was present (1) or absent (0). Finally, the filtered signals were assigned to a single class based on all possible fluorescent channel combinations. For instance, a vesicle with a positive signal in the 488 nm channel would be assigned to the [488] class. In contrast, a vesicle with positive signals in the 488 and 561nm channels would be assigned to the [488, 561] group, but not to the [488] class.

Supplementary Figures

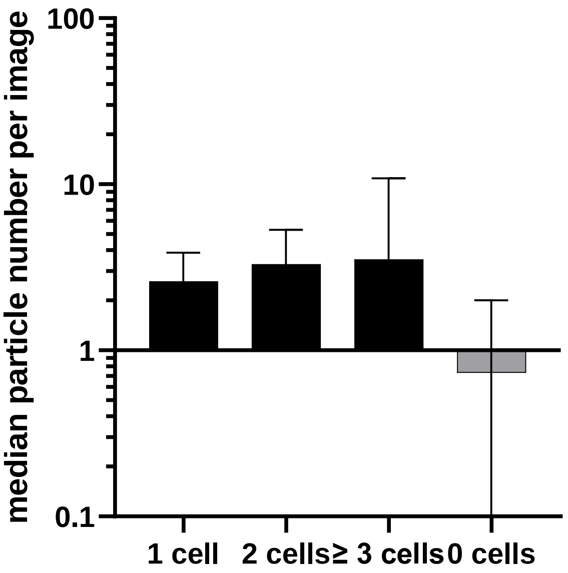

Figure SI1: Detecting EVs secreted from two, three or more cells. The relative frequency distribution shows increasing signal number of detected signals (a larger median) with increasing cell numbers compared to the control.

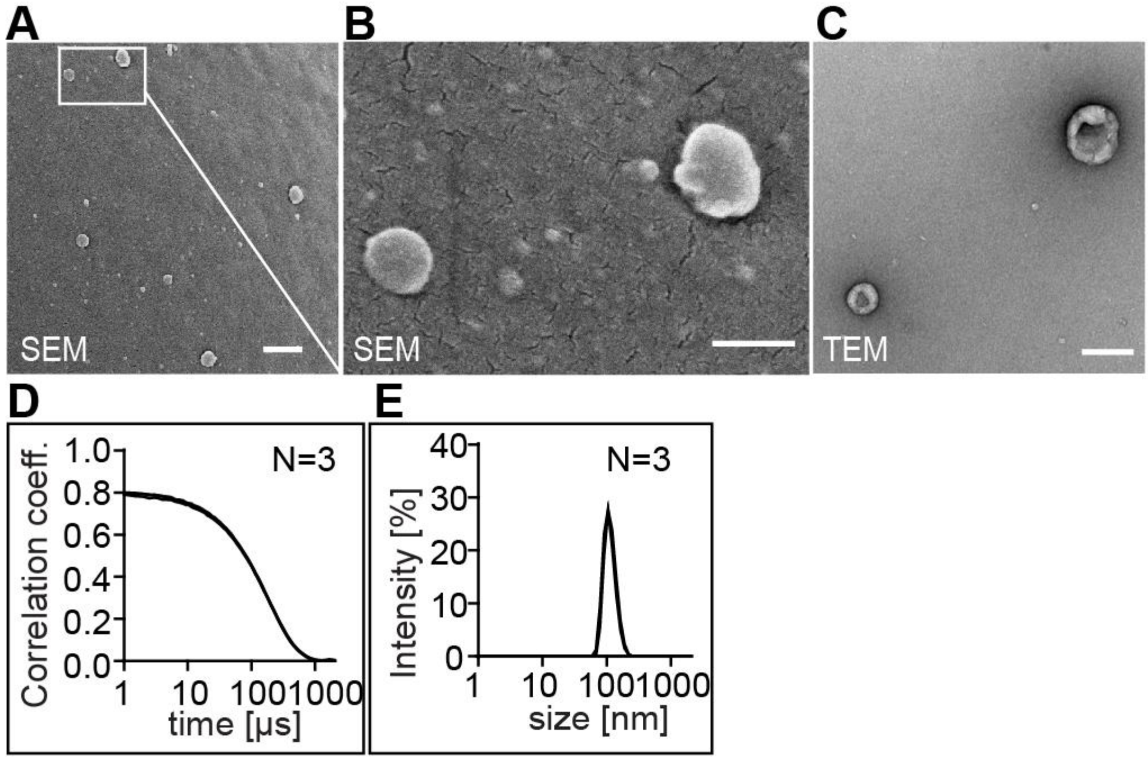

Figure SI2: Standard methods for EV detection provide only little and only morphological information about EVs. **A–C** Scanning and transmission electron microscopy images of immunochemically (anti-CD63) immobilized (A, B) or unspecifically immobilized (C) and negative stained EVs from differential ultracentrifuged mammalian cell culture supernatant on 16 µm-polystyrene beads (A, B) or copper grids C). Scale bars in (A) 400 nm, in (B) 100 nm, in (C) 150 nm. **D, E** Correlogram and intensity-size plot of dynamic light scattering measurements of EVs after differential ultracentrifugation in PBS. An intercept of 0.8 and higher and a smooth exponential decay reflects proper sample concentration without agglomerates. Ultracentrifuged EVs reflect a size-wise homogeneous, monomodal population with a theoretical hydrodynamic diameter of 100–200 nm. Both, electron microscopy as well as light scattering methods provide only high-resolution but biochemically unspecific but morphological information (SEM, TEM) or sample homogeneity.

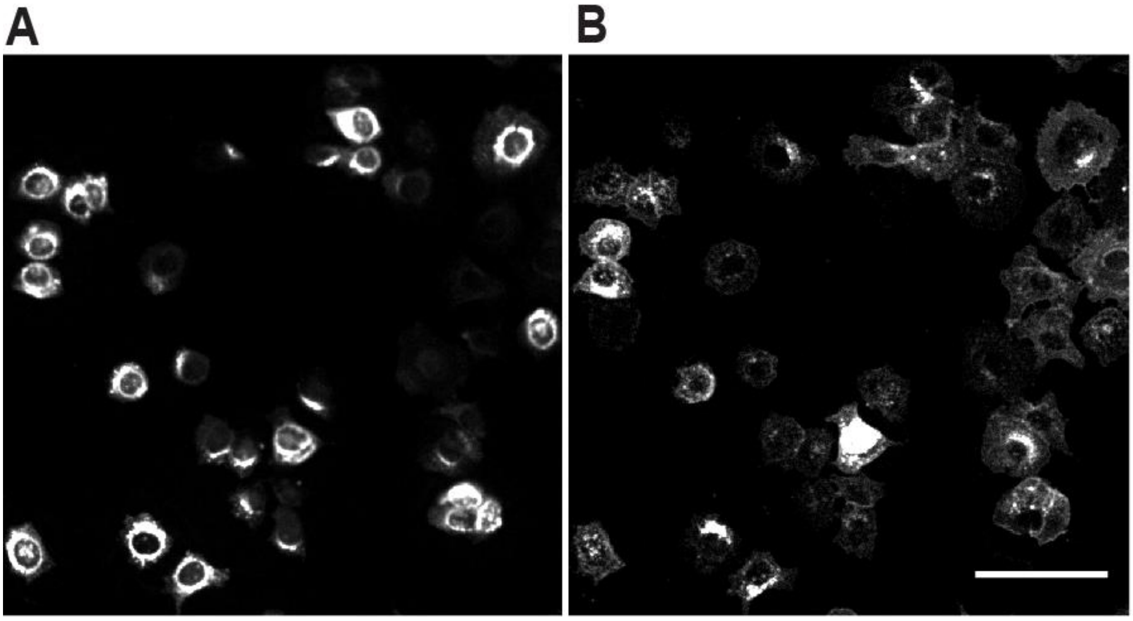

Figure SI3: Immunocytochemical staining of intracellular structure after paraformaldehyde fixation. Already without perforation (by e.g. Triton X-100, methanol) incubated MCF-7 cells can be intracellular stained after PFA fixation. **(A)** ANXA5 against cytosolic phosphatidylserines, enclosing the nuclei. **(B)** Anti-CD63 staining, present on intracellular vesicles (enclosing the nuclei) as well as outer plasma membrane. Scale bar 100 µm.

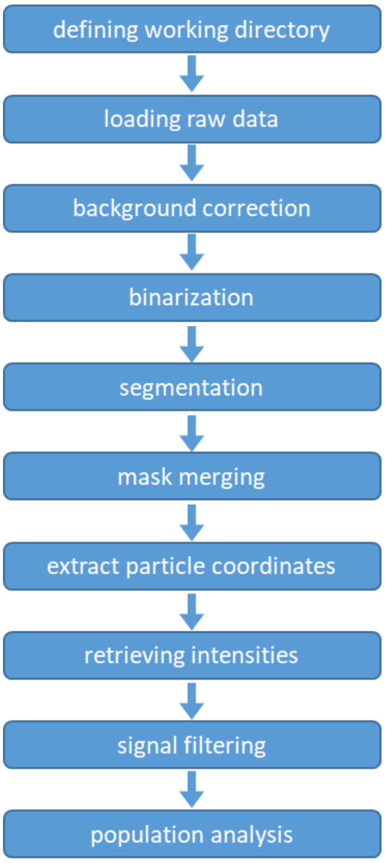

Figure SI4: Flow chart diagram of the image processing workflow. Acquired TIRFM images are loaded and background corrected. After background correction, signals are binarized and undergo segmentation steps, which include whole-filling, erosion, and dilating. After exclusion of particles > 1.2 µm, the masks are merged, and fluorescent signals are filtered. The workflow results in a binary attribute for every fluorescent channel, with either presence or absence of signals. Retrieved signals are then assigned to one of the fifteen possible populations.

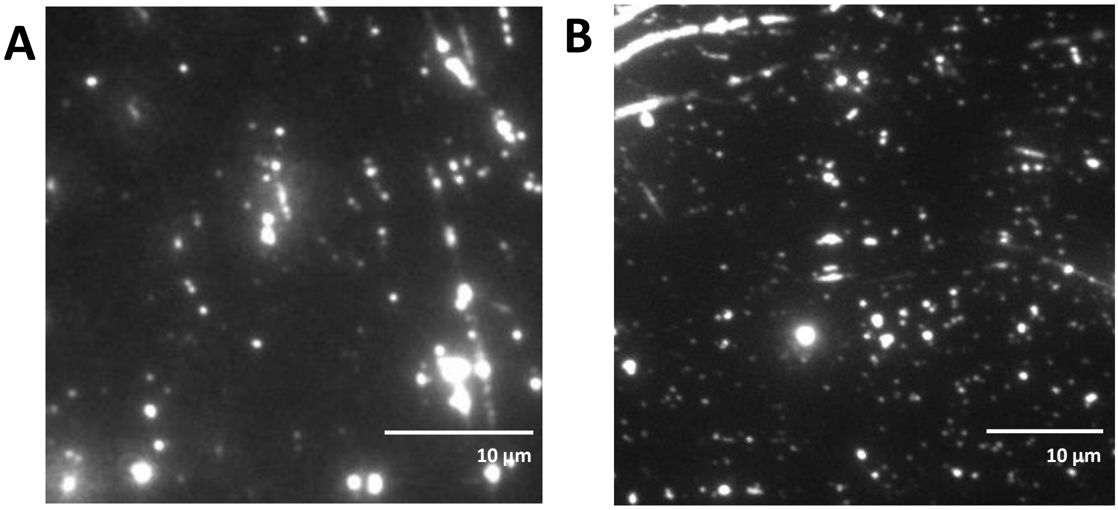

Figure SI5: Staining of EVs by the generic membrane dye DiO. TIRFM imaging revealed a large number of artifacts (aggregated dye or EVs, or changed EV morphology). To avoid artifacts due to potentially destructive impact of organic solvent used to dissolve the dye, or the dye itself, conjugates of fluorescent mAbs with a high specificity for a phenotype were applied instead of generic lipid dyes.

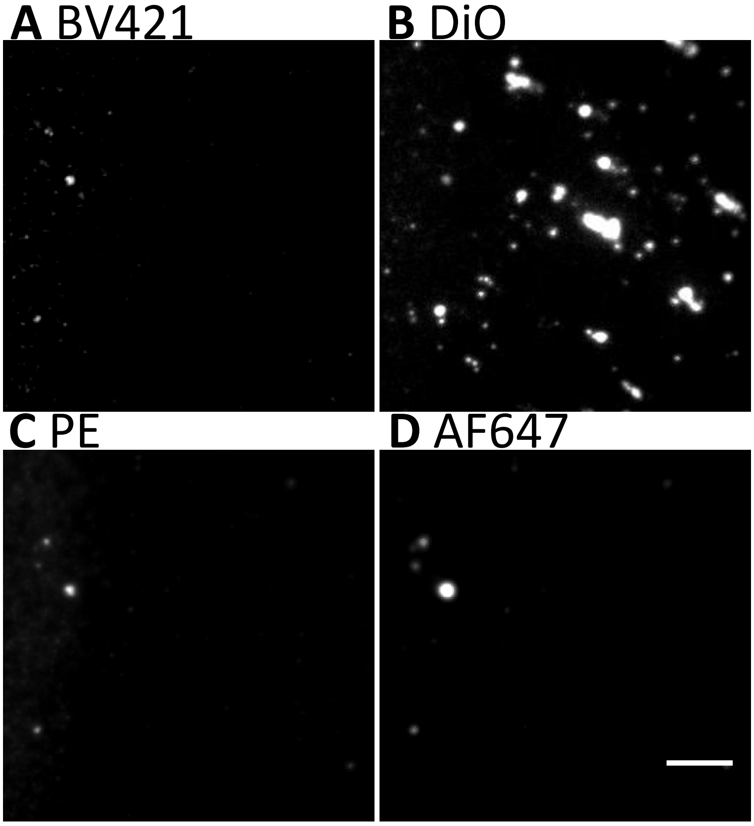

Figure SI6. Equivalent, background-corrected, 16-bit multicolor TIRFM images of DiO-stained EVs, as well as with the mAbs anti-CD63-BV421, anti-CD81-PE and ANXA5-AF647. All images have been treated equivalently for background correction. A) Fluorescent signals of Brilliant Violet 421 (BV421) with black point (BP) of 731 and white point (WP) of 27200 bits. B) DiO signals at BP/WP of 200/3500. The additional signals in this image originate from artifacts, e.g. increased background and labelled cell debris. C) PE signals at BP/WP 200/37814. D) AlexaFluor647-signals at BP/WP of 755/65535. Scale bar 5 μm.

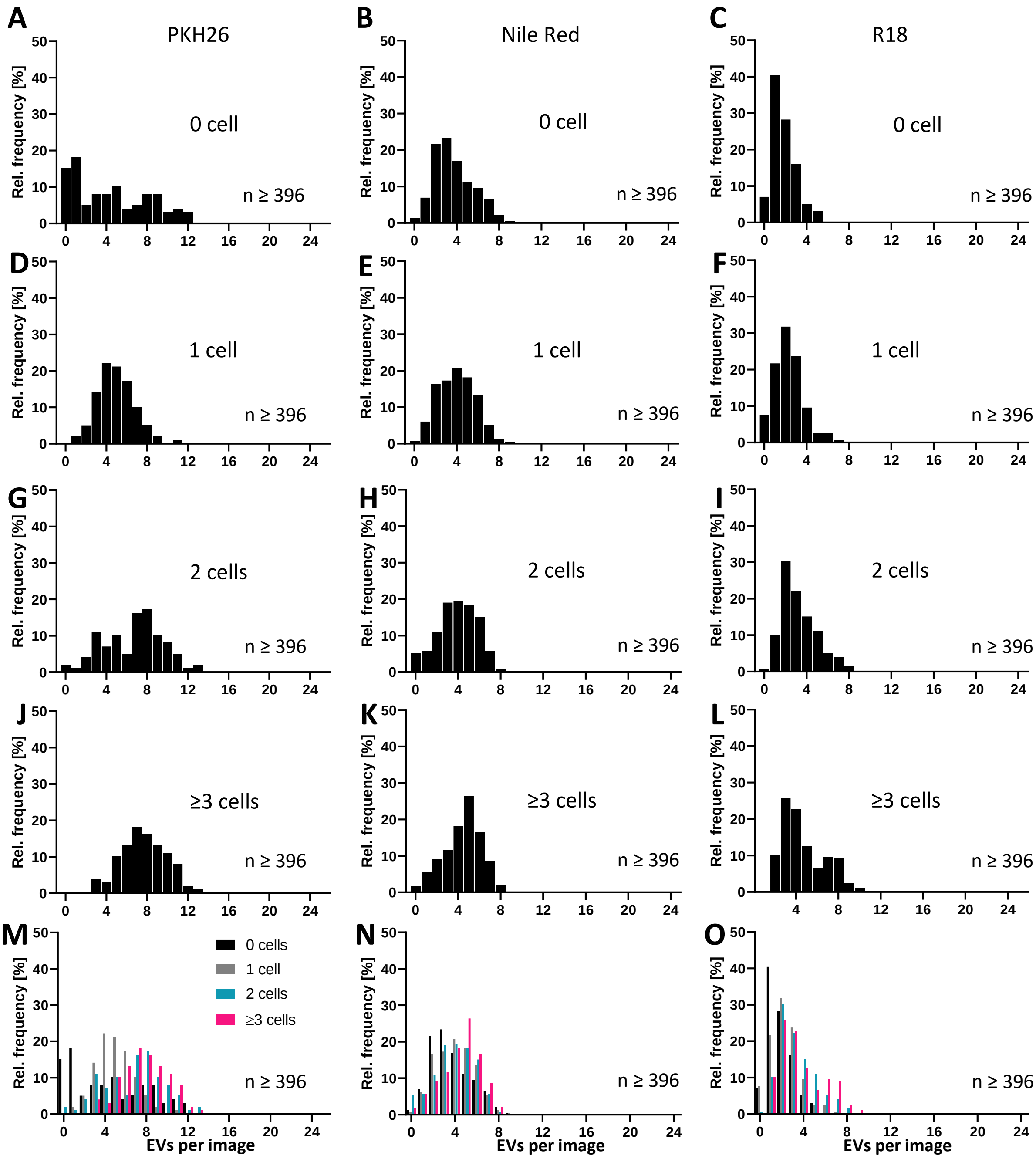
A
PKH26
0 cell
n ≥ 396
B
Nile Red
0 cell
n ≥ 396
C
R18
0 cell
n ≥ 396
D
1 cell
n ≥ 396
E
1 cell
n ≥ 396
F
1 cell
n ≥ 396
G
2 cells
n ≥ 396
H
2 cells
n ≥ 396
I
2 cells
n ≥ 396
J
≥3 cells
n ≥ 396
K
≥3 cells
n ≥ 396
L
≥3 cells
n ≥ 396
M
0 cells
1 cell
2 cells
≥3 cells
n ≥ 396
N
n ≥ 396
O
n ≥ 396
Rel. frequency [%]
EVs per image

Figure SI7. Relative frequency distributions of fluorescent signals in TIRFM representing imaged EVs of unoccupied wells (A-C) and wells occupied with 1 cell (D-F), 2 cells (G-I), and ≥3 cells (J-L) using the lipophilic dyes R18, Nile Red and PKH26. Figures M-O: overlaid histograms. The dyes contribute to a large (unspecific) background signal in the non-occupied control wells (A-C, compare to Fig. 4a in the manuscript). When filled with one or more cells (D-L, compare to Fig. 4c in the manuscript), the increase due to EVs formation and background signals cannot be differentiated, and the increase in EVs in wells that are occupied by cells cannot be quantified. Indeed, there seems to be an under-representation of low-intensity signals, as neither the empty nor the cell-containing wells show more than 12 EVs per image. In other words, strong signals are recognized as a single EV to the detriment of non-detected signals of less intensity. The problems of large background signals and clustering of EVs into high-intensity signals could be overcome by use of labelled mAb that bind specifically to proteins of the EVs.
